# Supplementary figures and images for: Dissection of a Complex Disease Susceptibility Region Using a Bayesian Stochastic Search Approach to Fine Mapping
Source: PLoS Genet. 2015 Jun 24;11(6):e1005272. doi: 10.1371/journal.pgen.1005272 (PMC4481316; doi:10.1371/journal.pgen.1005272)

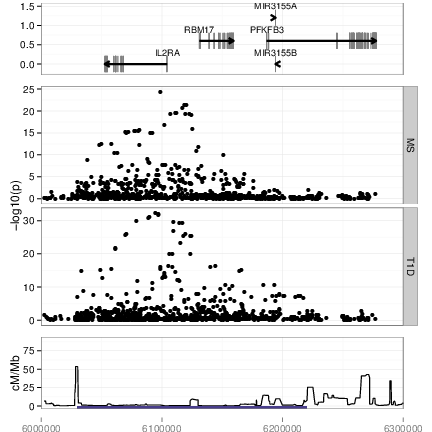

Supplement: S1 Fig — Bottom track shows HapMap recombination rates and the blue bar indicates the region targeted for fine mapping. (TIFF) [file pgen.1005272.s001.tiff]

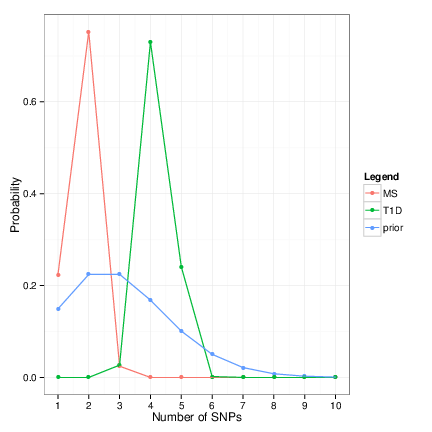

Supplement: S2 Fig — For posteriors, this is the sum of posterior probabilities over all models visited by GUESS which contain the number of SNPs shown. (TIFF) [file pgen.1005272.s002.tiff]

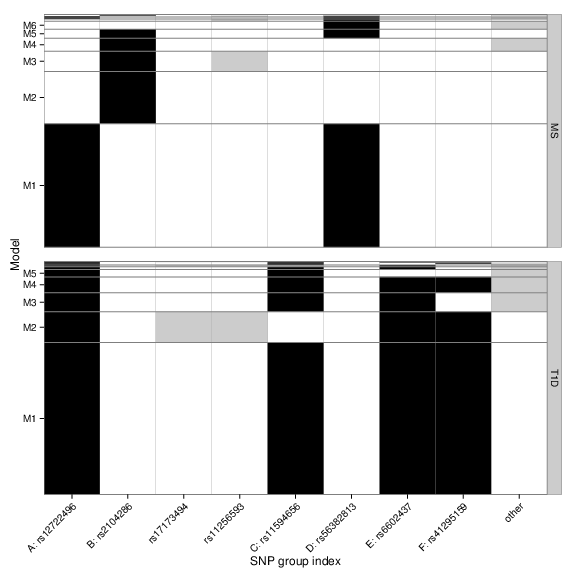

Supplement: S3 Fig — Total filled bar height is proportional to gMPPI for the group indexed by the SNP shown on the x axis. Only the most probable SNP groups (gMPPI > 0.1) are labelled; “other” denotes SNPs outside these more probable SNP groups. Black fill indicates high confidence SNP groups (A-F) that were taken forward for further analysis. Groups indexed by rs17173494 and rs11256593 were considered to have too little support for either disease for association to be declared with confidence. For MS, we see two competing models: M1 indexed by A (rs12722496) and D (rs56382813), and M2, indexed by B (rs2104286). (TIFF) [file pgen.1005272.s003.tiff]

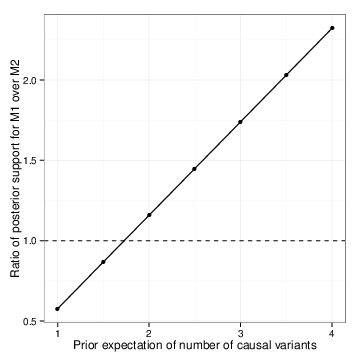

Supplement: S4 Fig — The relative posterior support is calculated as the posterior probability of all models within the M1 group divided by the posterior probability of all models within the M2 group for a given prior expectation. For a prior expectation of three, this is the ratio of bar heights for M1 over M2 from S4 Fig. (TIFF) [file pgen.1005272.s004.tiff]

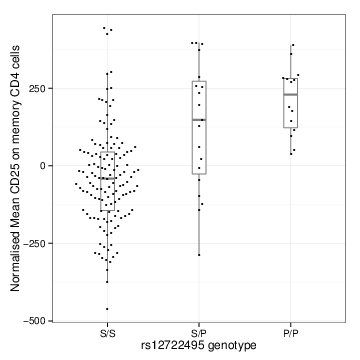

Supplement: S5 Fig — (TIFF) [file pgen.1005272.s005.tiff]

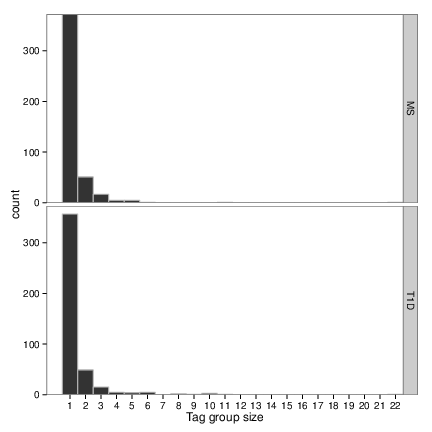

Supplement: S6 Fig — (TIFF) [file pgen.1005272.s006.tiff]

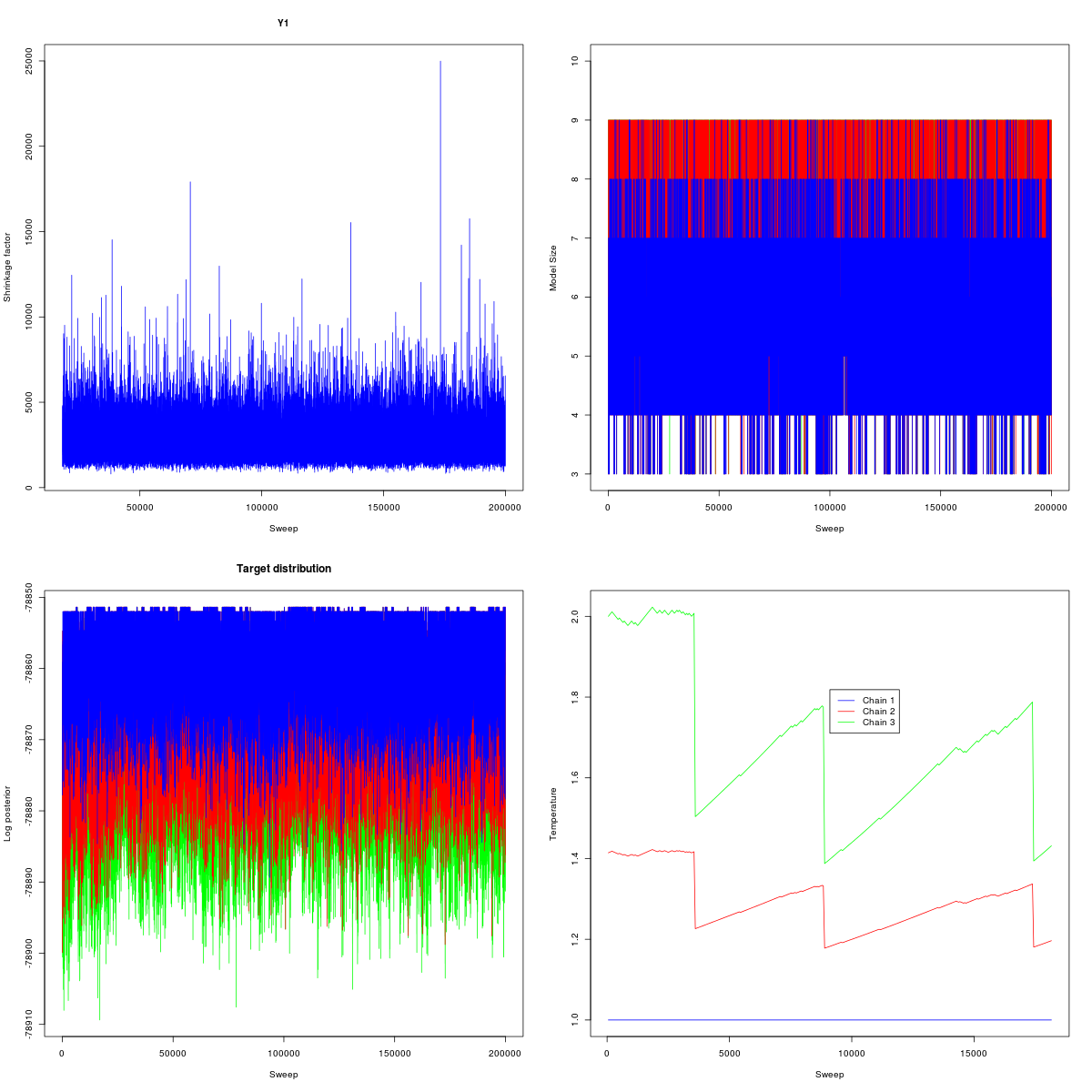

Supplement: S7 Fig — The three chains are indicated by different colours. (TIFF) [file pgen.1005272.s007.tiff]

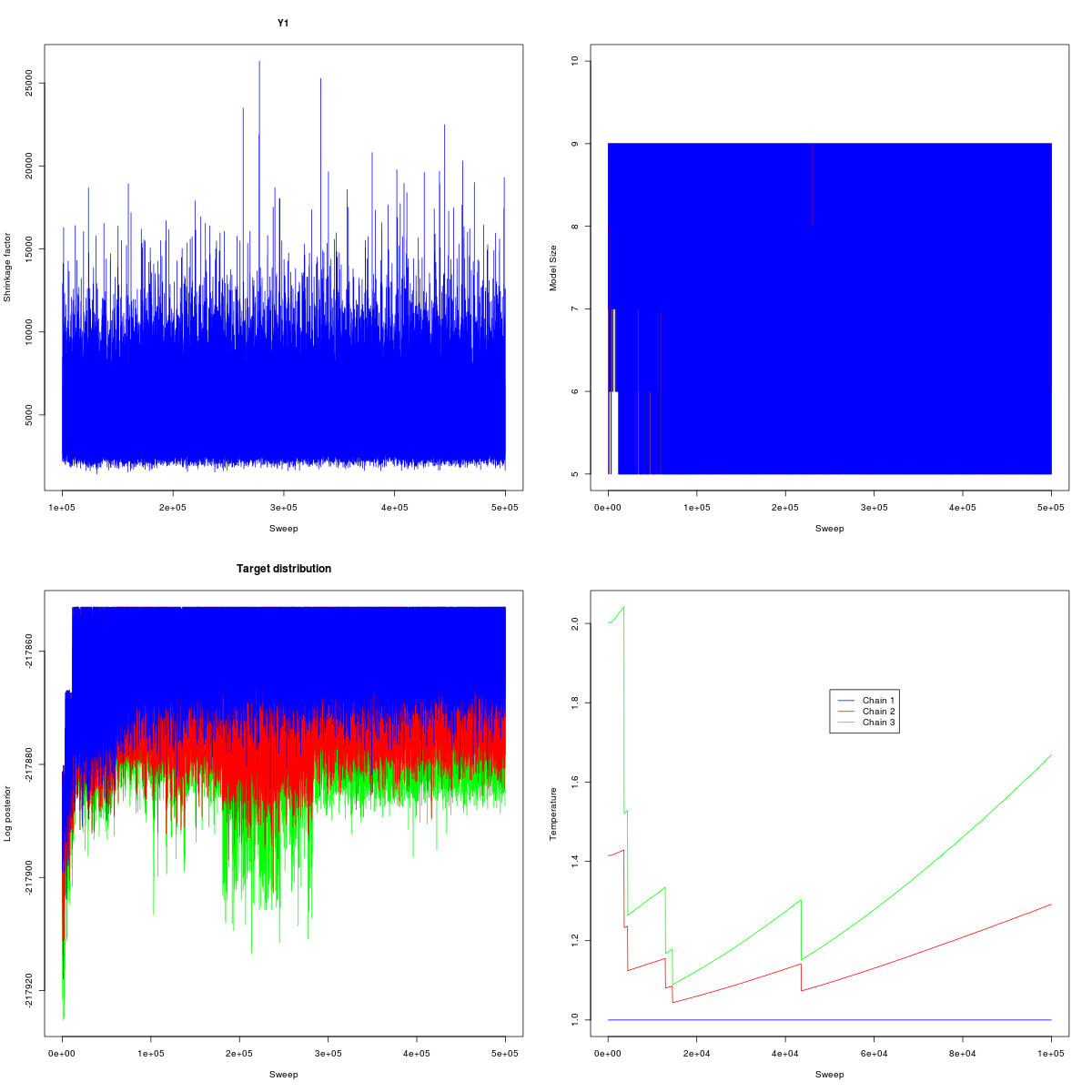

Supplement: S8 Fig — The three chains are indicated by different colours. (TIFF) [file pgen.1005272.s008.tiff]
