# Supplementary material for: Dissection of a Complex Disease Susceptibility Region Using a Bayesian Stochastic Search Approach to Fine Mapping
Source: PLoS Genet. 2015 Jun 24;11(6):e1005272. doi: 10.1371/journal.pgen.1005272 (PMC4481316; doi:10.1371/journal.pgen.1005272)
Supplement: S1 Table — (PDF) [file pgen.1005272.s009.pdf]

Trait	Country	Patients	Controls
T1D	UK	6693	12205
MS	Belgium	322	313
MS	Denmark	748	890
MS	Finland	466	345
MS	France	354	387
MS	Germany	1893	2621
MS	Italy	962	962
MS	Norway	701	911
MS	Sweden	1970	2121
MS	UK	9359	4542
MS	USA	2035	1837
MS	(all countries combined)	18810	14929
