# Supplementary material for: Dissection of a Complex Disease Susceptibility Region Using a Bayesian Stochastic Search Approach to Fine Mapping
Source: PLoS Genet. 2015 Jun 24;11(6):e1005272. doi: 10.1371/journal.pgen.1005272 (PMC4481316; doi:10.1371/journal.pgen.1005272)
Supplement: S2 Table — Briefly, we ran GUESSFM setting the mean expected number of causal variants per region to either three (GUESSFM-3) or five (GUESSFM-5). We calculated the discovery rate (the proportion of causal variants within at least one credible set, y axis) and false discovery rate (proportion of detected variants whose credible sets did not contain any causal variant, x axis) at different thresholds for the stepwise p value (< 10−6 or < 10−8), the group marginal posterior probability of inclusion (gMPPI > 0.5 or > 0.9) for GUESSFM and the regularization parameter λ (chosen to minimise the ten-fold cross validation error, or at the largest value that selected exactly three or five predictors) across simulated datasets. (PDF) [file pgen.1005272.s010.pdf]

Causal variants	Method	Criteria	Discovery rate	False discovery rate
2	GUESSFM-5	gMPPI> 0.5	0.966	0.009
2	GUESSFM-5	gMPPI> 0.9	0.939	0.003
2	GUESSFM-3	gMPPI> 0.5	0.970	0.015
2	GUESSFM-3	gMPPI> 0.9	0.943	0.004
2	Lasso	min CV error	0.994	0.982
2	Lasso	first 3 predictors	0.720	0.006
2	Lasso	first 5 predictors	0.880	0.015
2	Stepwise	$p < 10^{-6}$	0.719	0.004
2	Stepwise	$p < 10^{-8}$	0.644	0.003
2	Elastic net	min CV error	0.997	0.982
2	Elastic net	first 3 predictors	0.728	0.007
2	Elastic net	first 5 predictors	0.891	0.017
2	Grouped lasso	min CV error	0.986	0.947
2	Grouped lasso	first 3 predictors	0.671	0.021
2	Grouped lasso	first 5 predictors	0.846	0.044
3	GUESSFM-5	gMPPI> 0.5	0.900	0.011
3	GUESSFM-5	gMPPI> 0.9	0.797	0.002
3	GUESSFM-3	gMPPI> 0.5	0.900	0.021
3	GUESSFM-3	gMPPI> 0.9	0.833	0.005
3	Lasso	min CV error	0.992	0.974
3	Lasso	first 3 predictors	0.534	0.005
3	Lasso	first 5 predictors	0.692	0.013
3	Stepwise	$p < 10^{-6}$	0.552	0.009
3	Stepwise	$p < 10^{-8}$	0.458	0.005
3	Elastic net	min CV error	0.991	0.974
3	Elastic net	first 3 predictors	0.548	0.008
3	Elastic net	first 5 predictors	0.715	0.016
3	Grouped lasso	min CV error	0.964	0.937
3	Grouped lasso	first 3 predictors	0.481	0.019
3	Grouped lasso	first 5 predictors	0.648	0.040
4	GUESSFM-5	gMPPI> 0.5	0.856	0.023
4	GUESSFM-5	gMPPI> 0.9	0.747	0.008
4	GUESSFM-3	gMPPI> 0.5	0.801	0.033
4	GUESSFM-3	gMPPI> 0.9	0.690	0.014
4	Lasso	min CV error	0.995	0.967
4	Lasso	first 3 predictors	0.424	0.005
4	Lasso	first 5 predictors	0.567	0.011
4	Stepwise	$p < 10^{-6}$	0.440	0.011
4	Stepwise	$p < 10^{-8}$	0.340	0.007
4	Elastic net	min CV error	0.995	0.966
4	Elastic net	first 3 predictors	0.438	0.008
4	Elastic net	first 5 predictors	0.588	0.016
4	Grouped lasso	min CV error	0.928	0.931

Causal variants	Method	Criteria	Discovery rate	False discovery rate
4	Grouped lasso	first 3 predictors	0.385	0.018
4	Grouped lasso	first 5 predictors	0.530	0.037
5	GUESSFM-5	gMPPI> 0.5	0.755	0.033
5	GUESSFM-5	gMPPI> 0.9	0.635	0.008
5	GUESSFM-3	gMPPI> 0.5	0.715	0.038
5	GUESSFM-3	gMPPI> 0.9	0.589	0.014
5	Lasso	min CV error	0.993	0.958
5	Lasso	first 3 predictors	0.360	0.005
5	Lasso	first 5 predictors	0.494	0.011
5	Stepwise	$p < 10^{-6}$	0.346	0.014
5	Stepwise	$p < 10^{-8}$	0.252	0.007
5	Elastic net	min CV error	0.993	0.958
5	Elastic net	first 3 predictors	0.389	0.010
5	Elastic net	first 5 predictors	0.518	0.016
5	Grouped lasso	min CV error	0.911	0.918
5	Grouped lasso	first 3 predictors	0.326	0.017
5	Grouped lasso	first 5 predictors	0.455	0.036
