# Supplementary material for: Dissection of a Complex Disease Susceptibility Region Using a Bayesian Stochastic Search Approach to Fine Mapping
Source: PLoS Genet. 2015 Jun 24;11(6):e1005272. doi: 10.1371/journal.pgen.1005272 (PMC4481316; doi:10.1371/journal.pgen.1005272)
Supplement: S3 Table — All genes with measurable expression (reads > 20) in the studied samples are shown. Normalized read counts are shown for the two replicates of resting and stimulated CD4+ T cells. (PDF) [file pgen.1005272.s011.pdf]

Ensembl ID	Gene Symbol	Log_2 fold change	p	Read counts			
				Resting (1)	Resting (2)	Stimulated (1)	Stimulated (2)
ENSG00000134460	*IL2RA*	5.93	2.60^{-124}	189	294	9561	13644
ENSG00000170525	*PFKFB3*	1.85	1.63^{-15}	1372	1903	3881	5045
ENSG00000134453	*RBM17*	1.59	2.34×10^{-19}	1344	1495	3458	3138
